# Artificial cells eavesdropping on HepG2 cells

**DOI:** 10.1098/rsfs.2023.0007

**Published:** 2023-08-11

**Authors:** Isabella Nymann Westensee, Brigitte Städler

**Affiliations:** Interdisciplinary Nanoscience Center (iNANO), Aarhus University, Gustav Wieds Vej 14, 8000 Aarhus, Denmark

**Keywords:** artificial cell, cellular communication, cell mimicry, cytochrome P450

## Abstract

Cellular communication is a fundamental feature to ensure the survival of cellular assemblies, such as multicellular tissue, via coordinated adaption to changes in their surroundings. Consequently, the development of integrated semi-synthetic systems consisting of artificial cells (ACs) and mammalian cells requires feedback-based interactions. Here, we illustrate that ACs can eavesdrop on HepG2 cells focusing on the activity of cytochrome P450 1A2 (CYP1A2), an enzyme from the cytochrome P450 enzyme family. Specifically, d-cysteine is sent as a signal from the ACs via the triggered reduction of disulfide bonds. Simultaneously, HepG2 cells enzymatically convert 2-cyano-6-methoxybenzothiazole into 2-cyano-6-hydroxybenzothiazole that is released in the extracellular space. d-Cysteine and 2-cyano-6-hydroxybenzothiazole react to form d-luciferin. The ACs respond to this signal by converting d-luciferin into luminescence due to the presence of encapsulated luciferase in the ACs. As a result, the ACs can eavesdrop on the mammalian cells to evaluate the level of hepatic CYP1A2 function.

## 1. Introduction

Artificial cells (ACs) aim to mimic structural and functional features of mammalian cells or bacteria. The complexity of a living cell can be divided into its essential cellular hallmarks [1], such as compartmentalization [2–4], multicellular organization [5], energy generation [6,7], division [8], information processing and cellular communication [9,10]. ACs often aim to mimic a specific cellular hallmark rather than trying to imitate the entire complexity of the cellular machinery, resulting in simplified, synthetic models. The interaction of ACs with living cells and tissues is an important aspect that starts to be explored with the aim to interface the synthetic and natural world as discussed in more details by van Stevendaal *et al*. [11]. Interesting examples where ACs interact with or support their living counterparts include matrix vesicle-containing alginate microreactors to support biomineralization in SaOS-2 cell spheroids [12], lipid-based protocells producing a cytotoxin to induce cytotoxicity in cancer cells [13], platinum nanoparticle based microreactors to remove H_2_O_2_ and ${\mathrm{NH}}_{4}^{+}$ in a culture of primary rat cortical neurons to mitigate excitotoxicity [14], H_2_O_2_ scavenging microreactors co-cultured with hepatocytes in planar cell cultures or three-dimensional (3D) cell aggregates [15], T*β*4-exosome releasing microspheres that act as artificial stem cells to enhance the angiogenic capacity of coronary endothelial cells [16], or light-producing lipid-based synthetic cells to activate fungal cells [17].

The next step forward requires more interactivity between the different units, i.e. there should be a feedback loop or communication. This advancement aims to mimic cellular communication, which is essential to coordinate behaviour in living multicellular assemblies and tissue to adapt to changes in the surroundings and maintain overall homeostasis.

Communication has previously been demonstrated between different populations of ACs [18–22], or between ACs and living cells. The latter aspect has most widely been explored using bacterial cells for one-way [23,24] or reciprocal communication [25]. However, recent efforts started to explore the communication between ACs and mammalian cells. A one-way transfer of H_2_O_2_ from glucose oxidase-containing giant unilamellar vesicle as an AC model to red blood cells, which in response produce resorufin, has been demonstrated [26]. Further, one-way communication between genetically controlled ACs and neural stem cells was demonstrated, where the ACs produced a protein signal that led to stem cell differentiation [27]. We have shown a one-way signal transfer between HepG2 cells and alginate-based ACs functionalized with metalloporphyrins, which acted as mimics of cytochrome P450 (CYP) enzymes [28].

**Scheme 1.**
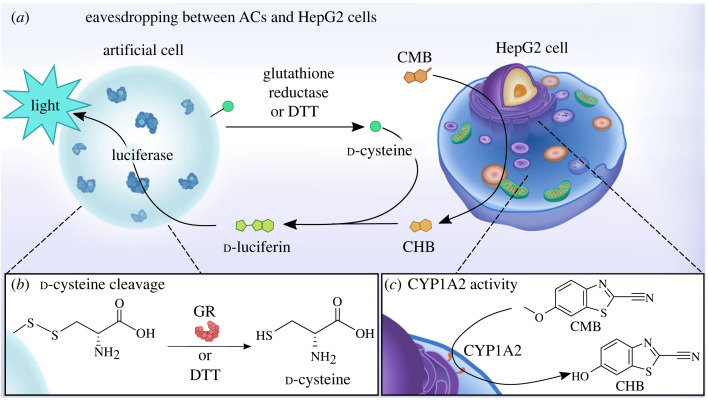
Reciprocal eavesdropping of ACs on HepG2 cells. (*a*) Schematic of the reciprocal eavesdropping. Alginate-based ACs are conjugated with d-cysteine that can be cleaved upon treatment with GR or DTT to reduce the disulfide bond between alginate and d-cysteine (*b*). CYP1A2 in HepG2 cells converts the luminometric compound CMB into CHB and (*c*) d-cysteine and CHB react to produce d-luciferin. d-Luciferin is converted by encapsulated luciferase to give a luminescence signal depending on the CYP1A2 expression level in HepG2 cells.

Herein, we demonstrated that ACs can ‘eavesdrop' on hepatocytes focusing on the expression level of cytochrome P450 1A2 (CYP1A2), an enzyme from the CYP enzyme family (scheme 1). Specifically, we functionalized alginate-based ACs with d-cysteine, which was released upon reduction of the linking disulfide bond with either glutathione reductase (GR) or dithiothreitol (DTT). d-Cysteine reacted with 2-cyano-6-hydroxybenzothiazole (CHB), which was produced by CYP1A2 catalysis in HepG2 cells and released to the extracellular space to create d-luciferin, which was converted to a luminescence signal by luciferase encapsulated in the ACs.

## 2. Experimental section

### 2.1. Materials

4-(2-Hydroxyethyl)piperazine-1-ethanesulfonic acid (HEPES, 99.5%), *N*-(3-dimethylaminopropyl)-*N*′-ethylcarbodiimide hydrochloride (EDC), dimethyl sulfoxide (DMSO), *N*-hydroxysuccinimide (NHS), poly(l-lysine) hydrobromide (PLL, M_w_ = 30–70 kDa), phosphate-buffered saline, 0.25% trypsin EDTA, calcium chloride dihydrate (CaCl_2_), adenosine 5′-triphosphate disodium salt hydrate (ATP, 99%), magnesium chloride hexahydrate (MgCl_2_), minimum essential medium Eagle (MEME, M2279, Sigma Aldrich), sodium pyruvate, 2-cyano-6-hydroxybenzothiazole (CHB, 776823), d-luciferin (L9504), luciferase from photinus pyralis (SRE0045), l-glutathione oxidized (G4376), glutathione reductase from baker's yeast (*S. cerevisiae*, G3664–500UN), dithiothreitol (DTT), nicotinamide adenine dinucleotide phosphate (NADPH, 10107824001), glutathione disulfide (GSSG), 5,5′-dithio-bis-(2-nitrobenzoic acid) (DTNB, Ellman's reagent), salicylamide, *S*-(2-pyridylthio)cysteamine hydrochloride, phosphate-buffered saline (PBS), clear round bottom 96-well plate ultra-low attachment, and sodium chloride were purchased from Sigma Aldrich.

Ultrapure low molecular weight sodium alginate with high α-l-guluronate (G block) (M_w_ < 75 kDa, min. 60% G block content, PRONOVA UP VLVG, Alg^L^) and ultrapure high molecular weight sodium alginate with high G block content (M_w_ > 200 kDa, min. 60% G block content, PRONOVA UP MVG, Alg^H^) were purchased from NovaMatrix. Fetal bovine serum (FBS), Dulbecco's Modified Eagle Medium (DMEM, high glucose, no glutamine, no methionine, no cysteine, 21013024), MEM non-essential amino acid solution, glutamine, streptomycin and penicillin, Pierce Dye Removal Columns, and DyLight 633 NHS Ester were purchased from Thermo Fisher Scientific. µ-Slide VI 0.4 (tissue culture treated, ibiTreat) was purchased from Ibidi. d-Cysteine (H62627.03), 2-cyano-6-methoxybenzothiazole (CMB, TCIAC1176), Corning cell strainers (40 µm and 100 µm, nylon), 96-well plate (white with clear bottom or clear), calcein-AM, and qualitative filter papers grade 600 (516–0309) were purchased from VWR.

HEPES buffer consisted of 10 mM HEPES and 150 mM NaCl at pH 7.4. Ultrapure water (18.2 MΩ cm^−1^ resistance) was provided by an ELGA Purelab Ultra system (ELGA LabWater, Lane End).

DMEM supplemented with 1% l-glutamine, 1% MEM non-essential amino acid solution, 1% Pen Strep and 3 mM salicylamide (prepared using a stock solution of 3 M salicylamide in DMSO) is referred to as l-cys free media.

### 2.2. Characterization of modified alginate

#### 2.2.1. Alg^cys^ synthesis

In total, 500 mg ultrapure high molecular weight sodium alginate with high G block content (Alg^H^) was functionalized according to a previously established protocol by Zelikin *et al*. [29]. Briefly, Alg^H^ was conjugated with a protected cysteamine, 2-pyridylthiocysteamine, and was subsequently reacted with d-cysteine at a basic pH to allow deprotonation of the thiol group and thiol–disulfide exchange, yielding Alg^Cys^ (electronic supplementary material, figure S1).

#### 2.2.2. Degree of functionalization of Alg^Cys^

37.5 mg ultrapure low molecular weight sodium alginate with high G block content (Alg^L^) and 37.5 mg Alg^H^ were dissolved in 5 ml ultrapure H_2_O to make a 15 mg ml^−1^ solution of Alg^L^/Alg^H^. 37.5 mg Alg^L^ and 37.5 mg Alg^Cys^ were dissolved in 5 ml ultarpure H_2_O to make a 15 mg ml^−1^ solution of Alg^L^/Alg^Cys^. Alginate mixtures were dissolved by rotating at room temperature for at least 2 h.

The degree of functionalization (DoF) of Alg^Cys^ was evaluated by treating Alg^L^/Alg^Cys^ with varying concentrations of DTT overnight and quantifying the amount of free thiols on the cleaved Alg^Cys^ using Ellman's reagent. 180 µl Alg^L^/Alg^H^ or Alg^L^/Alg^Cys^ were mixed with 20 µl DTT (0, 1, 10, 25 or 50 mM) in Eppendorf tubes for final DTT concentrations of 0, 0.1, 1, 2.5 or 5 mM. The samples were left overnight on a rotary shaker to allow for the reduction of the disulfide bond of Alg^Cys^. Then, the samples were washed by adding 100 µl sample and 400 µl PBS into a 10 kDa spin filter, and the samples were centrifuged at 12 × 10^3^ rpm for 15 min. This cleaning step was repeated 7× before the amount of free thiols present in the alginate mixtures (Alg^L^/Alg^H^ or Alg^L^/Alg^Cys^) and the filtrate was measured using Ellman's reagent. 5 µl thiol-containing sample, 70 µl PBS and 50 µl Ellman's reagent were added to a 96-well plate. A standard curve of d-cysteine (0–1 mM) was used to calculate the amount of free thiols. The plate was left for 2 min after the addition of Ellman's reagent to develop and the absorbance at 412 nm was measured. The amount of free thiols is plotted as the expected amount from 50 µl Alg^L^/Alg^H^ or Alg^L^/Alg^Cys^ to make it comparable to the experiments outlined in §§2.3.2, 2.5, 2.6.3 and 2.7.4.

#### 2.2.3. Activity of glutathione reductase

The activity of GR in either a KH_2_PO_4_ buffer (100 mM KH_2_PO_4_, 1 mM EDTA, pH 7) or l-cys free media was evaluated. First, stock solutions of GR, NADPH, DTNB and GSSG were prepared. A 6 U ml^−1^ solution of GR was prepared from the provided stock solution and subsequently, 15 µl 6 U ml^−1^ GR was added to 987 µl buffer for a GR solution of 0.09 U ml^−1^. 16 µl of an 80 mg ml^−1^ NADPH solution in ddH_2_O was added to 1 ml buffer or l-cys free media, resulting in a 1.26 mg ml^−1^ NADPH solution. 87.6 µl 3 mg ml^−1^ DTNB in DMSO was added to 912.4 µl buffer or l-cys free media to give a 0.26 mg ml^−1^ DTNB solution. A 6 mg ml^−1^ GSSG solution in buffer or l-cys free media was prepared. The assay was performed by adding the following components in the following order to a clear 96-well plate: 25 µl NADPH (1.26 mg ml^−1^), 50 µl GSSG (6 mg ml^−1^), 50 µl DTNB (0.26 mg ml^−1^) and 100 µl GR (0.09 U ml^−1^). 100 µl l-cys free media or buffer were used in the last step as the negative control. The reaction was monitored immediately after the addition of GR by measuring the absorbance at *λ*_abs_ = 412 nm every 10 s for 20 min.

#### 2.2.4. Cleavage of d-cysteine from Alg^Cys^

The amount of d-cysteine released from the alginate mixtures Alg^L^/Alg^H^ or Alg^L^/Alg^Cys^ was evaluated upon treatment with GR or DTT for 1 h at 37°C. CHB was added to react with released d-cysteine to form d-luciferin. 10 µl 15 mg ml^−1^ Alg^L^/Alg^H^ or Alg^L^/Alg^Cys^, 40 µl PBS, and 1 µl 5 mM CHB (50 µM final concentration) were added to Eppendorf tubes. Either (1) 49 µl l-cys free media, (2) 42.6 µl l-cys free media, 3.2 µl 25 U ml^−1^ GR (0.8 U ml^−1^ final concentration), and 3.2 µl 16 mg ml^−1^ NADPH (0.512 mg ml^−1^ final concentration), or (3) 45.8 µl l-cys free media and 3.2 µl 78 mM DTT (2.5 mM final concentration) were added to the Alg^L^/Alg^H^ or Alg^L^/Alg^Cys^ solutions. The Eppendorf tubes were left for 1 h at 37°C, before 50 µl was transferred to a white 96-well plate. 50 µl d-luciferin detection reagent (LDR) consisting of 0.5 mg luciferase, 10 mM MgCl_2_ and 120 µM ATP per ml (in PBS) was added to each well. The luminescence was read after 10 min using a PerkinElmer Enspire 2300 multilabel plate reader.

### 2.3. Assembly and characterization of artificial cells

#### 2.3.1. Assembly of artificial cells

Alginate beads were fabricated using an Encapsulator B-390 (Buchi). 5 ml of 15 mg ml^−1^ Alg^L^/Alg^H^ or Alg^L^/Alg^Cys^ solutions were loaded into a 20 ml syringe and connected to the Encapsulator B-390 through a plastic tube. The syringe was fixed onto a pump for a controlled injection speed of 1.1 ml min^−1^ through the Encapsulator B-390 with an inner nozzle diameter of 80 µm and an outer nozzle diameter of 120 µm. A frequency of 1300 Hz, an amplitude of 3, and an 800 V voltage were used during particle fabrication. The formed alginate particles were collected in a beaker containing 0.1 M CaCl_2_ solution while stirring and were allowed to cross-link for approximately 5 min at room temperature. The particles were then collected in a 40 µm cell strainer followed by rinsing with PBS buffer. Particles made from Alg^L^/Alg^H^ or Alg^L^/Alg^Cys^ solutions yielded AC^0^ or AC^Cys^, respectively. The ACs were visualized by using an inverted Olympus microscope (IX81), and the diameters were measured using ImageJ software. The size distribution was determined using Origin software (LogNormal fitting) and a minimum of 200 beads from three independent repeats were measured.

#### 2.3.2. Cleavage of d-cysteine from artificial cells

10 mg AC^0^ and AC^Cys^, 40 µl PBS and 1 µl 5 mM CHB were added to each well and the conditions of (1) no cleavage, (2) GR and (3) DTT were applied as outlined in §2.2.4 in the same volumes. The plate was left for 1 h at 37°C, 50 µl supernatant was transferred to a white 96-well plate, and 50 µl LDR was added to each well. The luminescence was read after 10 min using a PerkinElmer Enspire 2300 multilabel plate reader.

When plotting the data, the amount of obtained d-cysteine is given as the obtained amount per 50 mg AC^0^ or AC^Cys^. The statistical significance used to compare the means was determined using a one-way analysis of variance (ANOVA) followed by a Tukey's multiple comparison *post hoc* test.

### 2.4. Cell culture experiments

The human liver cancer cell line HepG2 was purchased from European Collection of Cell Cultures. HepG2 cells were cultured in 25 cm^2^ (TC-treated) culture flasks in MEME supplemented with 1% l-glutamine (2 mM), 1% MEM non-essential amino acid solution, 1% Pen Strep (100 µg ml^−1^ streptomycin and 100 U ml^−1^ penicillin) and 10% FBS (growth medium) at 37°C and 5% CO_2_. Experiments where ACs eavesdrop on HepG2 cells were performed in l-cys free media.

#### 2.4.1. Induction of HepG2 cells

CYP1A2 expression was induced in HepG2 cells using the aryl hydrocarbon receptor (AhR) agonists β-naphthoflavone (BNF) or indirubin [30]. HepG2 cells were seeded in a white 96-well culture plate at a final concentration of 30 × 10^3^ cells per well in 100 µl media. The well plates were kept in a humidified atmosphere at 37°C and 5% CO_2_ for either 24 h or 48 h. Then, the medium was removed and either new medium containing 0.5% (v/v) DMSO (no induction) or medium containing either 10 µM BNF or indirubin (1 µM or 10 µM final concentration, added at 0.5% (v/v) DMSO) was added to the wells. CYP1A2 expression was evaluated by adding the luminometric substrate CMB, which reacted with d-cysteine to form d-luciferin that subsequently was detected by the addition of luciferase.

Following the induction, wells were washed 2× with PBS, and the CYP1A2 activity was assessed by adding 49 µl l-cys free media supplemented with 3 mM salicylamide, 0.5 µl 5 mM CMB (in DMSO, for a final concentration of 50 µM) and 0.5 µl 10 mM d-cysteine (in ddH_2_O for a final concentration of 100 µM) to each well. The plate was left to incubate at 37°C and 5% CO_2_ for 1 h. 50 µl LDR was added to each well and the plate was read after 10 min. As controls, the addition of CMB, d-cysteine or ATP was omitted. A standard curve was made by preparing a dilution series of d-luciferin from 0 to 10 µM for the calculation of the amount of d-luciferin formed by the HepG2 cells.

### 2.5. Artificial cells eavesdropping on HepG2 cells in microslides

HepG2 cells were seeded at a final concentration of 30 × 10^3^ cells per µ-slide VI 0.4 (microslide). 100 µl of 30 × 10^4^ cells ml^−1^ was added to one chamber while tilted approximately 20°, and 60 µl was added to the second inlet chamber. The maximum volume of each chamber is 160 µl and each microslide consisted of six sets of neighbouring chambers. The microslides were placed in the incubator at 37°C in 5% CO_2_ while remaining tilted and the cells were allowed to adhere and grow at this tilted angle (electronic supplementary material, figure S2). New medium with 0.5% (v/v) DMSO (no induction) or 1 µM indirubin (at 0.5% v/v) was added to the cells after 24 h and 48 h. The next day, the chamber was washed 2× with PBS and 91 µl l-cys free media was added. 0.7 µl 10 mM CMB (final concentration 50 µM) was added to each chamber. Then, ACs, fabricated the day before, were added to the second inlet chamber by mixing 20 mg AC^0^ or AC^Cys^ with 40 µl l-cys free media. Next, either (1) 9.0 µl l-cys free media (no cleavage), (2) 4.5 µl 25 U ml^−1^ GR, 4.5 µl 16 mg ml^−1^ NADPH or (3) 4.5 µl 78 mM DTT and 4.5 µl l-cys free media were added and the microslides were left for 2 h at 37°C and 5% CO_2_. Next, 50 µl medium was transferred to a white 96-well plate, 50 µl freshly prepared LDR was added to each well, and the luminescence was read after 10 min using a PerkinElmer Enspire 2300 multilabel plate reader. A standard curve was prepared by mixing 0–10 µM CHB with 300 µM d-cysteine for 2 h at 37°C and 5% CO_2_, followed by transferring 25 µl to a white 96-well plate, and adding 25 µl PBS and 50 µl LDR. A standard curve was prepared with each experiment to evaluate the amount of CHB formed from HepG2 cells. The statistical significance used to compare the means was determined using a one-way ANOVA followed by a Tukey's multiple comparison *post hoc* test.

### 2.6. Artificial cells eavesdropping on HepG2 cells in aggregates

#### 2.6.1. Fabrication of artificial cells used in aggregates

ACs were fabricated as described in §2.3.1. After collection in the 40 µm cell strainer, the ACs were filtered through a 100 µm cell strainer and the ACs smaller than 100 µm were collected. Poly-l-lysine (PLL) was conjugated with rhodamine B isothiocyanate (R) as previously described [31]. The ACs were coated with PLL^R^ by mixing 1 ml 50 mg ml^−1^ AC solution (in HEPES buffer) with 1 ml 1.2 mg ml^−1^ PLL^R^ solution (in HEPES buffer) in a 1 : 1 ratio. The solution was left to incubate for 1 h at 4°C to allow coating of the ACs with PLL^R^. Subsequently, the solution was washed 3× with 0.1 M CaCl_2_ solution by spinning the solution at 4.5 × 10^3^ rpm for 5 min. The resulting 50 mg PLL^R^ coated ACs were resuspended in 1 ml growth medium. The solution was diluted 5× into PBS buffer, and the concentration was determined by manually counting all beads in 3 × 2 µl of solution. The ACs were visualized by taking bright field and fluorescence microscopy images with a U-MNG filter cube (Olympus) for detecting PLL^R^ using an inverted Olympus microscope (IX81). The diameters of a minimum of 200 beads from three independent repeats were measured on bright field microscopy images using ImageJ software, and the size distribution was determined using Origin software (LogNormal fitting).

#### 2.6.2. Aggregate formation and characterization

AC^0^ or AC^Cys^ with PLL^R^ coating were co-cultured with HepG2 cells at a cell-to-AC ratio of 32:1 in 96-well plates with an ultralow attachment surface. 20 000 HepG2 cells and 606 ACs were used per aggregate in 200 µl growth medium. A stock solution of 30.3 × 10^3^ ACs per ml (in growth medium) was prepared and 20 µl was added to each well. A stock solution of 40 × 10^3^ HepG2 cells per ml was prepared and 100 µl was added to each well, followed by 80 µl growth medium. The aggregates were left to form for 3 days, and the growth medium was exchanged on day 3 and 4 to growth medium with or without 1 µM indirubin (final concentration). After 48 h induction, on day 5, the eavesdropping experiment was performed (see §2.6.3).

The aggregates were visualized by taking bright field and fluorescence microscopy images with a U-MNG filter cube (Olympus) for detecting PLL^R^ using an inverted Olympus microscope (IX81). Aggregates exposed to GR or DTT were imaged and compared as well as untreated aggregates. Further, aggregates treated with DTT were visualized using confocal laser scanning microscopy (CLSM) with a Zeiss LSM700 CLSM (Carl Zeiss, Germany). A staining solution of 4 µM calcein-AM in PBS was prepared, and the aggregates were stained for 10 min. Then, the aggregates were transferred using a 1 ml pipette to a glass slide for imaging with CLSM using a 20× objective. Tile scans were obtained with a resolution of 512 × 512 pixels, and dimensions of 5 × 5 or 3 × 3. Cleaved calcein-AM in live cells was imaged using *λ*_ex_/*λ*_em_ = 488/493–532 nm, and PLL^R^ was visualized using *λ*_ex_/*λ*_em_ = 555/560–800 nm.

#### 2.6.3. AC^Cys^ eavesdropping on HepG2 cells in aggregates

The eavesdropping experiment was performed after growing the AC/HepG2 aggregates for 5 days, including 2 days of induction. Then, the aggregates were washed 2× with PBS and 186.2 µl l-cys free media was added. Next, 1 µl 10 mM CMB (final concentration 50 µM), and either (1) 12.8 µl l-cys free media (no cleavage), (2) 6.4 µl 25 U ml^−1^ GR and 6.4 µl 16 mg ml^−1^ NADPH, or (3) 6.4 µl 78 mM DTT and 6.4 µl l-cys free media were added and the well plate was left for 2 h at 37°C and 5% CO_2_. Next, 50 µl media was transferred to a white 96-well plate and 50 µl LDR was added to each well. The luminescence was read after 10 min using a plate reader. A standard curve of CHB was prepared with each experiment as outlined in §2.5. The statistical significance used to compare the means was determined using a one-way ANOVA followed by a Tukey's multiple comparison *post hoc* test.

### 2.7. Luciferase-loaded AC^Cys^ eavesdropping on HepG2 cells

#### 2.7.1. Labelling of luciferase with DyLight 633 (Luc^F^)

Luciferase was fluorescently labelled using DyLight 633 Amine-Reactive Dye. The NHS ester-activated derivative of DyLight 633 was used to label the free amines on luciferase. First, 0.5 mg of luciferase was dissolved in 125 µl HEPES (4 mg ml^−1^, pH 7.4). DyLight (5 µg ml^−1^ in 125 µl HEPES) was mixed 1:1 (v/v) with luciferase for a final concentration of 2.5 µg ml^−1^ and 2 mg ml^−1^ luciferase, respectively. The Eppendorf was left to rotate for 5 h at room temperature. Then, the non-conjugated dye was removed by purification on a resin fluorescent dye removal column. The column was prepared as per the manufacturer's protocol. Briefly, the dye removal resin was mixed thoroughly and 250 µl was transferred to a spin column. The column was centrifuged for 30 s at 1000×*g* to remove the storage solution, the collection tube discarded, and the resin column placed in a new collection tube. 250 µl of the labelling reaction solution with luciferase (2 mg ml^−1^) and DyLight 633 was transferred to the resin and was pipetted up and down to mix thoroughly. Then, the column was centrifuged for 30 s at 1000×*g*, and the purified protein (Luc^F^, 2 mg ml^−1^) was collected as the filtrate.

#### 2.7.2. Encapsulation and imaging of luciferase-loaded AC^Cys^

Alg^L^/Alg^Cys^ was dissolved at a concentration of 18.75 mg ml^−1^ in HEPES buffer under rotation at room temperature for approximately 2 h. Luciferase, labelled or non-labelled, was encapsulated in AC^Cys^. Non-labelled luciferase was dissolved at a concentration of 2 mg ml^−1^ in 500 µl HEPES and was diluted 1 : 4 in the 18.75 mg ml^−1^ Alg^L^/Alg^Cys^ solution for a final luciferase concentration of 0.4 mg ml^−1^ and a final concentration of Alg^L^/Alg^Cys^ of 15 mg ml^−1^. Labelled luciferase was encapsulated by mixing 250 µl 2 mg ml^−1^ Luc^F^ with 250 µl 2 mg ml^−1^ non-labelled luciferase. The final volume of 500 µl 2 mg ml^−1^ luciferase was mixed with 2 ml 18.75 mg ml^−1^ Alg^L^/Alg^Cys^ solution.

The solutions were mixed well and alginate particles were fabricated as previously described in §2.3.1, resulting in ${\mathrm{AC}}_{\mathrm{Luc}}^{\mathrm{Cys}}$ or ${\mathrm{AC}}_{\mathrm{LucF}}^{\mathrm{Cys}}$. The latter assemblies were used to fabricate ACs for CLSM imaging using *λ*_ex_/*λ*_em_ = 639/644–800 nm.

#### 2.7.3. Preparation of paper chips

The paper chips were prepared using a previously published protocol [32]. The diameter of the circular paper chips was chosen to be 45 mm to fit into a 96-well plate with a diameter of 5 mm, and the width of the printed ring was 1.1 mm. The pattern on the circular paper chips was designed in Microsoft Powerpoint and was printed onto both sides of an A0 VWR qualitative filter paper grade 600 (516–0309) cut to an A4 size, using a LaserJet printer (Xerox WorkCentre 7845). The printed sheets were autoclaved to melt the ink particles that resulted in a hydrophobic barrier that allows the paper chip to float on aqueous solutions. The individual circular paper chips were cut out under sterile conditions and applied to the top of the wells with cultured cells in a 96-well plate using a tweezer.

#### 2.7.4. Artificial cell eavesdropping in paper chips on HepG2 cells

HepG2 cells were seeded in at a final concentration of 30 × 10^3^ cells per well in 100 µl growth medium in a white 96-well plate, and the cells were allowed to settle overnight at 37°C in 5% CO_2_. New growth medium with 0.5% (v/v) DMSO (no induction) or 1 µM indirubin (at 0.5% v/v) was added to the HepG2 cells after 24 h and 48 h. The next day, the chamber was washed 2× with PBS and 91 µl l-cys free media was added. Paper chips were added to each well using tweezers and 20 mg ACs were added to each paper chip. Next, 0.5 µl 10 mM CMB (final concentration 50 µM) and either (1) 6.4 µl l-cys free media (no cleavage), (2) 3.2 µl 25 U ml^−1^ GR and 3.2 µl 16 mg ml^−1^ NADPH or (3) 3.2 µl 78 mM DTT and 3.2 µl l-cys free media were added directly to the media underneath the paper chip. The well plate was left for 2 h at 37°C and 5% CO_2_. Next, 100 µl of a 10 mM MgCl_2_ and 120 µM ATP solution (in PBS) was added to each well and the luminescence was read after 10 min using a plate reader. A standard curve of CHB was prepared with each experiment as outlined in §2.5.

Further, wells were prepared without seeded HepG2 cells. Instead, 50 µM CHB (final concentration at 0.5% (v/v) DMSO) was added to each well to evaluate the amount of released d-cysteine upon reduction. The statistical significance used to compare the means was determined using a one-way ANOVA followed by a Tukey's multiple comparison *post hoc* test.

## 3. Results and discussion

### 3.1. Fabrication of artificial cells with d-cysteine as a cleavable signal

First, ACs capable of eavesdropping on HepG2 cells require the ability to send a signal on demand. Alginate was chosen as the structural scaffold of the ACs, since it is a naturally derived polysaccharide offering good biocompatibility. Hydrogels have recently gained interest as a structural scaffold in the fabrication of ACs since they replicate the gel-like properties of the intracellular environment in mammalian cells as reviewed recently by Allen *et al*. [33]. Alginate in particular was successfully employed for AC assembly [12,15,34], including for an one-way signal transfer from ACs to HepG2 cells [28].

We used the carboxylic acid on alginate for functionalization by first conjugating 2-pyridylthiocysteamine before adding d-cysteine via thiol–disulfide exchange (electronic supplementary material, figure S1). As a result, d-cysteine conjugated to high molecular weight alginate (Alg^H^) through a disulfide bond was obtained (Alg^Cys^; electronic supplementary material, figure S3a), which upon mixing 1:1 (v/v) with low molecular weight alginate yielded a solution of Alg^L^/Alg^Cys^. The DoF of Alg^Cys^ was determined by treating the Alg^L^/Alg^Cys^ solution with varying concentrations of DTT overnight to reduce the disulfide bond. The amount of free thiols was quantified using Ellman's reagent after washing the reduced Alg^L^/Alg^Cys^, which corresponded to a DoF of 5.6% on Alg^Cys^ (electronic supplementary material, figure S3bi). Importantly, the pristine alginate solution Alg^L^/Alg^H^ yielded no detectable thiols, and no excess DTT was found in the filtrate. Further, when treating Alg^L^/Alg^Cys^ with 2.5 mM DTT for 1 h, the amount of d-cysteine in the filtrate was measured by incubation with CHB, and the produced d-luciferin was measured by adding luciferase and ATP (electronic supplementary material, figure S3bii). Luminescence was only detected in the Alg^L^/Alg^Cys^ filtrate, and not from Alg^L^/Alg^H^, showing that d-cysteine was released upon treatment with DTT for 1 h.

Next, the enzyme GR from *S. cerevisiae* was used to catalyse the reduction of the disulfide bond using NADPH as an electron donor, as a more biologically relevant alternative to the small molecule DTT. The activity of GR was found to be approximately 45% lower in cell media than in K_2_HPO_4_ + EDTA buffer (pH 7.5) probably due to difference in salt composition or the presence of calcium (electronic supplementary material, figure S3c).

Following on, Alg^L^/Alg^H^ and Alg^L^/Alg^Cys^ dissolved in l-cys free media were treated with either 0.8 U mL^−1^ GR or 2.5 mM DTT for 2 h at 37°C to release the d-cysteine. Alg^L^/Alg^H^ and Alg^L^/Alg^Cys^ incubations in l-cys free media were used as controls. The released d-cysteine was incubated with CHB in excess for 30 min to produce d-luciferin, which was quantified by monitoring the signal obtained from its conversion by luciferase into luminescence. 50 µl Alg^Cys^ released approximately 25 µM and 150 µM d-cysteine when treated with GR and DTT, respectively (electronic supplementary material, figure S3d), illustrating the expected ability of GR to release d-cysteine but with lower efficacy than DTT. Alg^Cys^ is not the natural substrate for GR, which can explain the lower efficacy of the enzyme to reduce the disulfide bond compared to the non-specific DTT. Alg^L^/Alg^H^ gave no detectable signal and a small amount (approx. 5 µM) of passively released d-cysteine was detected for untreated Alg^L^/Alg^Cys^.

**Figure 1.**
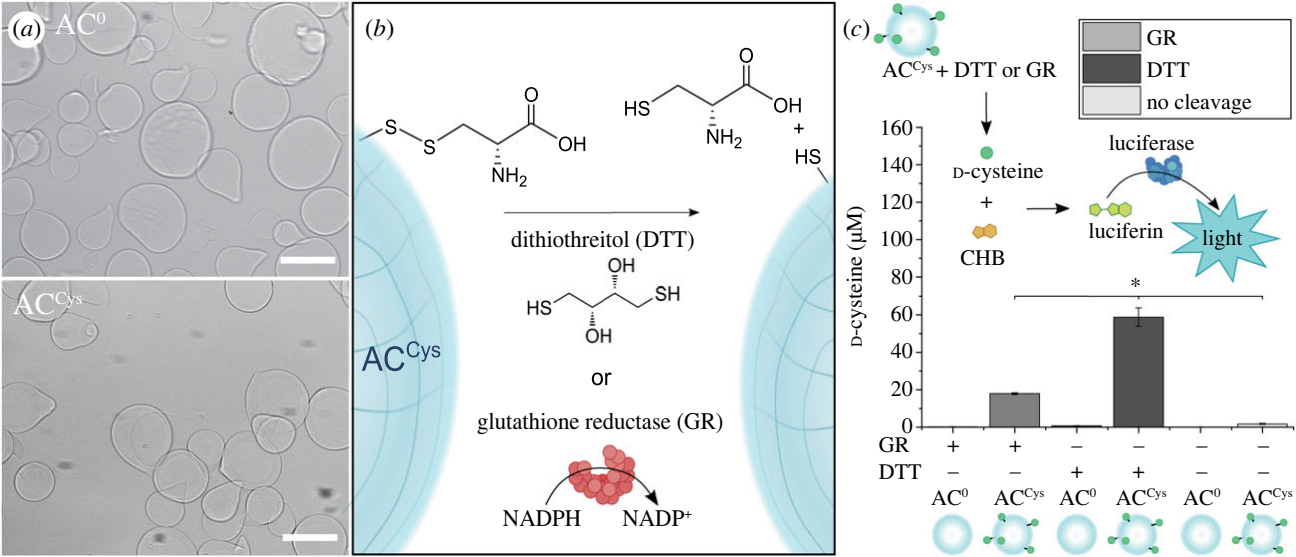
Characterization of alginate beads conjugated with d-cysteine (AC^Cys^). (*a*) Bright field microscopy images of AC^0^ and AC^Cys^. Scale bar is 100 µm. (*b*) d-Cysteine is conjugated to alginate in AC^Cys^ and DTT or GR is used to reduce the disulfide bond to release d-cysteine. (*c*) Quantification of d-cysteine after treating AC^0^ or AC^Cys^ with GR or DTT. 50 mg AC^Cys^ yielded approximately 60 µM d-cysteine after 1 h incubation with 2.5 mM DTT (*n* = 3). *p < 0.05, determined using a one-way ANOVA followed by a Tukey's multiple comparison *post hoc* test.

Alg^L^/Alg^H^ and Alg^L^/Alg^Cys^ were then used to make microgels to act as ACs capable of eavesdropping on HepG2 cells. The microgels were fabricated employing either Alg^L^/Alg^H^ or Alg^L^/Alg^Cys^ with the extrusion dripping method using an Encapsulator B390, followed by cross-linking in a calcium bath resulting in AC^0^ and AC^Cys^, respectively, with diameters of approximately 90 µm (figure 1*a*; electronic supplementary material, figure S4). The amount of d-cysteine released from 50 mg AC^Cys^ (equivalent of approx. 50 µl Alg^L^/Alg^H^ or Alg^L^/Alg^Cys^) was found to be 20 µM and 60 µM upon exposure to GR and DTT, respectively, while no released d-cysteine was detected from AC^0^.

### 3.2. Eavesdropping of artificial cells on two-dimensional cultured HepG2 cells

The next step was to evaluate the activity of HepG2 cells towards the conversion of the compound CMB that was shown to be dominantly dependent on CYP1A2 [35]. CYP activity was chosen in this project to report on hepatic activity due to its importance in drug metabolism. This is a core task carried out by hepatocytes in the liver, which is achieved through phase I and II metabolism, whereof the most common phase I reaction is oxidation carried out by CYPs [36]. HepG2 cells generally show lower basal gene expression levels of CYPs compared to, for example, primary human hepatocytes, but CYP1A2 has previously been shown to be inducible in HepG2 cells with AhR agonists [30]. We employed 1 µM indirubin as a suitable AhR agonist for induction, and this compound was therefore used for all subsequent experiments (for details, see electronic supplementary material, figure S5 and the related text).

**Figure 2.**
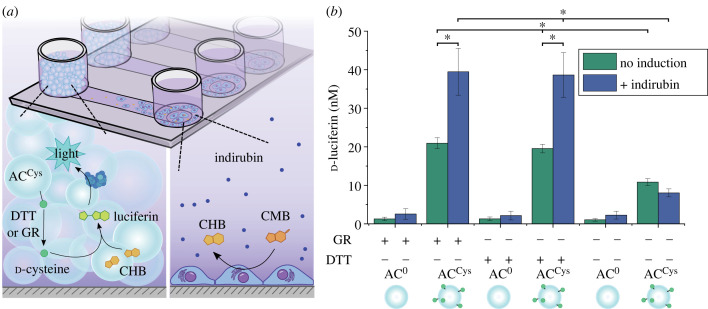
Eavesdropping in microslides. (*a*) CYP1A2 expression is induced in HepG2 cells using indirubin. CYP1A2 converts CMB to CHB through dealkylation. CHB reacts with d-cysteine provided by the AC^Cys^ to yield d-luciferin, which is converted by luciferase to produce luminescence. (*b*) Determined CHB production of HepG2 cells based on the detected amount of d-luciferin (*n* = 3). *p < 0.05, determined using a one-way ANOVA followed by a Tukey's multiple comparison *post hoc* test.

With the aim to illustrate that the ACs could eavesdrop on mammalian cells, AC^Cys^ and HepG2 cells were added to two spatially separated inlet chambers on the same microslide, which were connected through a central channel to allow for exchange of molecules by diffusion. The HepG2 cells were seeded in an inlet chamber and left to attach for 24 h followed by induction with indirubin for 48 h before the addition of AC^Cys^ to the second inlet chamber. CMB and either GR or DTT were added to the chamber with the HepG2 cells for 2 h. The resulting d-luciferin was detected by addition of luciferase (figure 2*a*). The amount of d-luciferin was proportional to the amount of CHB produced by the HepG2 cells, since the ACs yielded d-cysteine in excess in the µM range (figure 1*c*). Pristine and induced HepG2 cells resulted in a significant difference in d-luciferin, with levels of approximately 20 nM and approximately 40 nM, respectively (figure 2*b*), demonstrating that the AC^Cys^ can eavesdrop on significant changes in the CHB production. Notably, GR and DTT usage for d-cysteine cleavage from AC^Cys^ yielded d-luciferin levels that were not significantly different, showing that both methods of cleavage yield an excess of d-cysteine. Further, the d-luciferin levels corresponded to the amount of CHB produced by HepG2 cells when d-cysteine was added externally to the solution rather than being supplied by AC^Cys^ (electronic supplementary material, figure S5b). This aspect showed that the presence of AC^Cys^ did not affect the CYP activity, but served to eavesdrop on the HepG2 cells. Importantly, the use of AC^0^ yielded background levels of luminescence expected in the absence of d-cysteine. Similarly, omitting CMB and luciferase also only resulted in background luminescent signals (electronic supplementary material, figure S6). However, around approximately 10 nM d-luciferin was detected when AC^Cys^ were exposed to neither DTT nor GR, which likely reflected a small amount of spontaneously released d-cysteine as this level was lower than the amount of available CHB in solution.

### 3.3. Eavesdropping of artificial cells on HepG2 in three-dimensional aggregates

**Figure 3.**
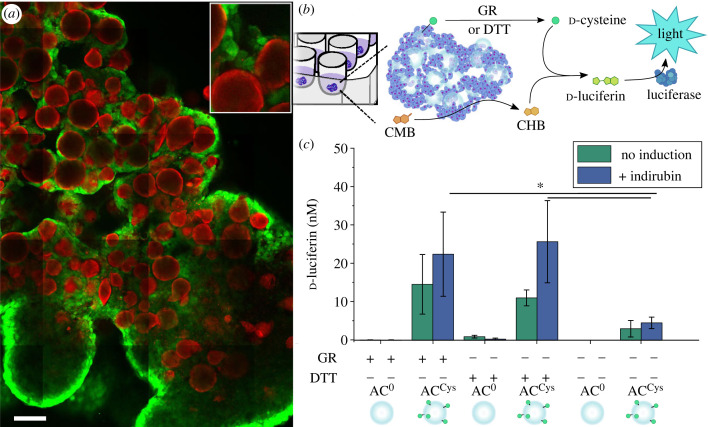
Eavesdropping in semi-synthetic aggregates. (*a*) Representative CLSM images of aggregates consisting of HepG2 cells and AC^0^ grown for 5 days. Calcein-AM is used to visualize the live cells (green) and the AC^0^s are coated with PLL^R^ (red). Scale bar is 100 µm. (*b*) Cartoon showing the eavesdropping of AC^Cys^ on the HepG2 cells in the aggregates. (*c*) d-Luciferin made from CHB produced by HepG2 cells and d-cysteine cleaved from AC^Cys^ (*n* = 4). *p < 0.05, determined using a one-way ANOVA followed by a Tukey's multiple comparison *post hoc* test.

The direct interaction between ACs and mammalian cells in aggregates is a first step towards the assembly of 3D multicellular functional semi-synthetic tissue. Thus, the aim was to demonstrate the eavesdropping capability of AC^Cys^ when cultured with HepG2 cells into semi-synthetic aggregates. Here, alginate beads were fabricated as before using the extrusion dripping method with an added step of filtration through a 100 µm cell strainer to remove larger beads that would less readily integrate with the HepG2 cells likely due to their fast sedimentation to the bottom of the culture wells. This filtration step shifted the average diameter from approximately 90 µm (electronic supplementary material, figure S4) to approximately 45 µm (electronic supplementary material, figure S7a,b) for AC^0^ and AC^Cys^. The beads were then coated with rhodamine B isothiocyanate conjugated PLL (PLL^R^) for visualization and to render the beads positively charged to promote cell adhesion. The PLL^R^ coating led to wrinkling of the surface of the particles, which was more pronounced for AC^Cys^ than for AC^0^ (electronic supplementary material, figure S7c) with no noticeable change in size (electronic supplementary material, figure S7d). This observation was most likely due to the lower cross-linking density in AC^Cys^, which had fewer carboxylic acid groups available since they were used for d-cysteine conjugation. The HepG2 cells and AC^Cys^ were seeded in a 1:32 AC^Cys^:HepG2 cell ratio in ultralow adhesion surface round-bottom plates, and left to incubate for 2 days to allow for aggregate formation. Then, the aggregates were induced with indirubin for 2 days before imaging. Bright field microscopy images of the semi-synthetic aggregates revealed that the HepG2 cells formed a dense ring in the periphery and a higher degree of AC^Cys^ present in the centre of the aggregates (electronic supplementary material, figure S8). The semi-synthetic aggregates were imaged using CLSM after incubation with calcein-AM to visualize the live HepG2 cells (figure 3*a*). There were no observable differences in aggregate formation or HepG2 cell viability depending on the presence of AC^Cys^ or AC^0^ due to differences in surface morphology and alginate modification. 3D projection of *z*-stacks further showed the successful formation of semi-synthetic aggregates with HepG2 cells growing around AC^Cys^ and AC^0^ (electronic supplementary material, figure S9). Further, treatment with GR or DTT led to no apparent changes in the appearance of the semi-synthetic aggregates; in particular, DTT treatment did not decrease the fluorescence from the calcein-AM staining (electronic supplementary material, figure S10), suggesting no adverse effect on cell viability.

First, the amount of d-cysteine released from AC^Cys^ in the aggregates was quantified since fewer ACs were used to fabricate cell aggregates (approx. 600 ACs per aggregate) compared with the amount of ACs used for the eavesdropping in the microchannels (20 mg). The amount of d-cysteine released from the AC^Cys^ in the aggregates in the presence of excess CHB (50 µM) was approximately 61 nM and 1.53 µM when incubated with GR and DTT, respectively (electronic supplementary material, figure S11). This d-cysteine amount was significantly lower compared to the eavesdropping experiments in the microslides due to the low amount of AC^Cys^ present in the aggregates. Additionally, the aggregate environment might present a diffusion limitation. However, since the amount of CHB converted from HepG2 cells was the limiting step for the eavesdropping in microslides (approx. 40 nM; figure 2*b*), enough d-cysteine was expected to be released from AC^Cys^. In order to prevent any potential impact on the yield of d-luciferin under the reaction conditions used in the eavesdropping experiments (2 h at 37°C and 5% CO_2_), a range of concentrations of d-cysteine (10–500 nM) was mixed with 0–100 nM CHB to identify the detection limits in buffer solutions (electronic supplementary material, figure S12). For a given CHB concentration, luminescence read-outs were comparable when using d-cysteine concentrations of 500 nM, 100 nM, or 50 nM, with the detection limit for CHB being 2 nM. However, lower luminescence was observed at stoichiometric CHB concentrations when using 25 nM or 10 nM d-cysteine. Therefore, the approximately 61 nM d-cysteine cleaved from the AC^Cys^ in the aggregates using GR was sufficient for eavesdropping. However, lower yields would present a limitation from the AC^Cys^ in supplying sufficient d-cysteine for the eavesdropping. The d-cysteine yield could be improved by using DTT, increasing the AC^Cys^ to HepG2 cell ratio, conjugating higher amounts of d-cysteine to alginate, using higher amounts of Alg^Cys^ for the AC^Cys^ assembly, or a combination thereof.

Following on, the HepG2 cells in the aggregates were induced with indirubin for 2 days before CMB and GR or DTT were added for 2 h. The resulting d-luciferin was detected by the addition of luciferase and monitoring of the luminescence (figure 3*b*). Similar levels of d-luciferin were produced when AC^Cys^-containing aggregates were exposed to GR and DTT for d-cysteine cleavage, which were statistically significantly higher compared to untreated aggregates. There was a trend of higher amounts of produced d-luciferin in aggregates with induced HepG2 cells compared with pristine HepG2 cells, but the difference was not statistically significant. DTT treatment led to approximately 10 nM and 25 nM d-luciferin without and with indirubin treatment, respectively. This outcome was approximately 2× lower than when performing eavesdropping in microslides. However, the amount of CHB (based on the detected d-luciferin) obtained from induced cells, when differences in the amount of seeded cells were accounted for, was approximately 1.25 × 10^−3^ nM per cell and 1.3 × 10^−3^ nM per cell in aggregates and microslides, respectively. Therefore, comparable results were obtained between the two experimental set-ups. As expected, the use of AC^0^ in the aggregates yielded no detectable luminescence signal. Aggregates with untreated AC^Cys^ resulted in approximately 5 nM d-luciferin, which was approximately 2× lower than in the microslides, suggesting less AC^Cys^ being present in the former case. However, this level was higher than expected with the low amount of AC^Cys^ present in the aggregates, which might be due to the direct interaction with the HepG2 cells in the aggregates leading to an increased non-triggered d-cysteine release.

### 3.4. Reciprocal eavesdropping between artificial cells and HepG2 cells

**Figure 4.**
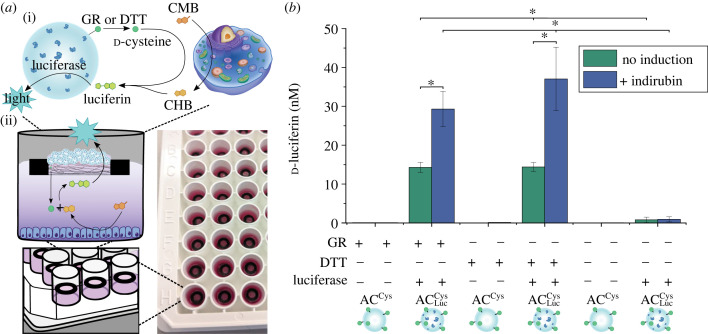
(*a*) (i) Reciprocal eavesdropping of ${\mathrm{AC}}_{\mathrm{Luc}}^{\mathrm{Cys}}$ on HepG2 cells. (ii) Schematic showing the experimental set-up. (*b*) d-Luciferin production as a result of CHB made by HepG2 cells and cleavage of d-cysteine from ACs. *p < 0.05, determined using a one-way ANOVA followed by a Tukey's multiple comparison *post hoc* test.

Finally, the goal was to demonstrate that the ACs could not only send the signal (d-cysteine), but also receive the response (d-luciferin) and reply (produce luminescence) (figure 4*a*(i)). Therefore, luciferase was encapsulated in AC^Cys^ resulting in ${\mathrm{AC}}_{\mathrm{Luc}}^{\mathrm{Cys}}$ (electronic supplementary material, figure S13a). Further, the experimental set-up was adapted to ensure that the encapsulated enzyme remained active, i.e. too long incubation times were avoided. In this set-up, a floating paper chip, which we have previously characterized and used in mammalian cell culture [32], was added to each well of a 96-well plate and 20 mg of ${\mathrm{AC}}_{\mathrm{Luc}}^{\mathrm{Cys}}$ was added in the middle of each paper chip (figure 4*a*(ii)).

First, the activity of ${\mathrm{AC}}_{\mathrm{Luc}}^{\mathrm{Cys}}$ on the paper chips was evaluated and compared to ${\mathrm{AC}}_{\mathrm{Luc}}^{\mathrm{Cys}}$ in solution to determine if luciferase in ${\mathrm{AC}}_{\mathrm{Luc}}^{\mathrm{Cys}}$ remained active, and if the paper chip hindered the reaction (electronic supplementary material, figure S13b). The luminescence signal, which was required to calculate the d-cysteine release, was obtained by adding either GR or DTT to the ${\mathrm{AC}}_{\mathrm{Luc}}^{\mathrm{Cys}}$ on the paper chips and supplementing the bulk solution with excess CHB. A significant difference of approximately 1.4 µM and 5.8 µM d-cysteine was detected when GR and DTT were used, respectively. By contrast, ${\mathrm{AC}}_{\mathrm{Luc}}^{\mathrm{Cys}}$ in solution resulted in approximately × 2 to × 3 higher amount of released d-cysteine; however, the difference was only significant when using DTT. This illustrated that the paper chips hindered the cleavage reaction either by acting as a diffusion barrier or limiting the mixing of ${\mathrm{AC}}_{\mathrm{Luc}}^{\mathrm{Cys}}$ with the bulk solution. However, the d-cysteine levels obtained from ${\mathrm{AC}}_{\mathrm{Luc}}^{\mathrm{Cys}}$ on the paper chips were expected to be sufficient for the eavesdropping.

Second, the reciprocal eavesdropping of ${\mathrm{AC}}_{\mathrm{Luc}}^{\mathrm{Cys}}$ on HepG2 cells was demonstrated. To this end, HepG2 cells were seeded and induced with indirubin in a white 96-well plate before the ${\mathrm{AC}}_{\mathrm{Luc}}^{\mathrm{Cys}}$-containing paper chips were added. GR or DTT was added for 2 h, and the resulting luminescence signal was monitored as a measure for the amount of produced d-luciferin (or produced CHB). Approximately 30 µM d-luciferin was detected for the induced HepG2 cells, while statistically significant lower amounts of d-luciferin (approx. 14 µM) were measured in the pristine HepG2 cells, illustrating that the luminescent signal reflected the CHB release by the HepG2 cells, i.e. successful eavesdropping was demonstrated (figure 4*b*). A trend towards a higher yield of d-luciferin when using DTT (approx. 37 µM) compared to GR (approx. 29 µM) was observed, which might be due to the diffusion barrier presented by the paper chip to GR resulting in slower or lower d-cysteine release, but the difference was not statistically significant.

## 4. Conclusion

Taken together, eavesdropping of ACs on the CYP1A2 activity in HepG2 cells was demonstrated by detecting differences in pristine and induced HepG2 cells.

The eavesdropping capability of the ACs illustrates an easily implemented approach to monitor a core hepatic function when co-cultured with hepatocytes. Using ACs instead of bulk assays offers opportunities to bring reaction molecules into close proximity to the partner units or spatio-temporal control of molecule synthesis and release, among others. The next step forward will be the implementation of ACs capable of reciprocal communication with mammalian cells with the goal to influence the behaviour of both the ACs and the living cells.

## Data Availability

Data are available from the Dryad Digital Repository: https://doi.org/10.5061/dryad.2rbnzs7sf [37]. The data are provided in electronic supplementary material [38].
